# Joint radial trajectory correction for accelerated T_2_^*^ mapping on an MR‐Linac

**DOI:** 10.1002/mp.16479

**Published:** 2023-05-27

**Authors:** Wajiha Bano, Will Holmes, Rosie Goodburn, Mohammad Golbabaee, Amit Gupta, Sam Withey, Alison Tree, Uwe Oelfke, Andreas Wetscherek

**Affiliations:** ^1^ Joint Department of Physics The Institute of Cancer Research and The Royal Marsden NHS Foundation Trust London UK; ^2^ Department of Engineering Mathematics University of Bristol Bristol UK; ^3^ The Royal Marsden NHS Foundation Trust and The Institute of Cancer Research London UK

**Keywords:** gradient delay correction, hypoxia imaging, MR‐Linac, T_2_^*^ mapping

## Abstract

**Background:**

T_2_^*^ mapping can characterize tumor hypoxia, which may be associated with resistance to therapy. Acquiring T_2_^*^ maps during MR‐guided radiotherapy could inform treatment adaptation by, for example, escalating the dose to resistant sub‐volumes.

**Purpose:**

The purpose of this work is to demonstrate the feasibility of the accelerated T_2_^*^ mapping technique using model‐based image reconstruction with integrated trajectory auto‐correction (TrACR) for MR‐guided radiotherapy on an MR‐Linear accelerator (MR‐Linac).

**Materials and methods:**

The proposed method was validated in a numerical phantom, where two T_2_^*^ mapping approaches (sequential and joint) were compared for different noise levels (0,0.1,0.5,1) and gradient delays ([1, ‐1] and [1, ‐2] in units of dwell time for x‐ and y‐axis, respectively). Fully sampled k‐space was retrospectively undersampled using two different undersampling patterns. Root mean square errors (RMSEs) were calculated between reconstructed T_2_^*^ maps and ground truth. In vivo data was acquired twice weekly in one prostate and one head and neck cancer patient undergoing treatment on a 1.5 T MR‐Linac. Data were retrospectively undersampled and T_2_^*^ maps reconstructed, with and without trajectory corrections were compared.

**Results:**

Numerical simulations demonstrated that, for all noise levels, T_2_^*^ maps reconstructed with a joint approach demonstrated less error compared to an uncorrected and sequential approach. For a noise level of 0.1, uniform undersampling and gradient delay [1, ‐1] (in units of dwell time for x‐ and y‐axis, respectively), RMSEs for sequential and joint approaches were 13.01 and 9.32 ms, respectively, which reduced to 10.92 and 5.89 ms for a gradient delay of [1, 2]. Similarly, for alternate undersampling and gradient delay [1, ‐1], RMSEs for sequential and joint approaches were 9.80 and 8.90 ms, respectively, which reduced to 9.10 and 5.40 ms for gradient delay [1, 2]. For in vivo data, T_2_^*^ maps reconstructed with our proposed approach resulted in less artifacts and improved visual appearance compared to the uncorrected approach. For both prostate and head and neck cancer patients, T_2_^*^ maps reconstructed from different treatment fractions showed changes within the planning target volume (PTV).

**Conclusion:**

Using the proposed approach, a retrospective data‐driven gradient delay correction can be performed, which is particularly relevant for hybrid devices, where full information on the machine configuration is not available for image reconstruction. T_2_^*^ maps were acquired in under 5 min and can be integrated into MR‐guided radiotherapy treatment workflows, which minimizes patient burden and leaves time for additional imaging for online adaptive radiotherapy on an MR‐Linac.

## 1 INTRODUCTION

The availability of hybrid MR‐Linacs,^1^, ^2^ which combine an MRI system with a linear accelerator, enables daily MR imaging of patients with cancer over a course of radiotherapy. MRI's potential to identify hypoxic volumes within the tumor is of particular interest for radiotherapy, because such regions are associated with therapy resistance and may vary throughout treatment.^3^ Characterizing daily changes in tumor hypoxia minutes before treatment delivery could inform adaptive dose escalation to hypoxic sub‐volumes to improve patient outcomes. Quantitative MRI (qMRI)‐based biomarkers allow for non‐invasive assessment of morphological, biological, and functional processes in tissue, as well as response to radiotherapy.^4^ One such qMRI parameter, the transverse relaxation time T_2_^*^, is sensitive to the concentration of paramagnetic deoxyhemoglobin within the vascular compartment of tissues.^5^ Previous studies have shown a correlation between the associated relaxation rate R_2_^*^ (R_2_^*^ = 1/T_2_^*^) and invasive hypoxia measurements using needle electrodes and immunohistochemical staining.^6^, ^7^ Furthermore, increased tumor hypoxia indicated by R_2_^*^ maps has been demonstrated in patients with prostate cancer following androgen deprivation therapy.^8^ The clinical feasibility of measuring cyclic tumor oxygenation through quantification of R_2_^*^ was shown in head and neck tumors and a median oscillation period of 15 min was reported.^3^

In current MR‐guided radiotherapy workflows on MR‐Linacs, there is a time window when the MRI system is not being used, while delineations of volumes of interest are updated to the daily anatomy and re‐planning is performed. Such “opportunity time” could be utilized to acquire quantitative MRI, including T_2_^*^ mapping, without prolonging the overall treatment time. However, implementing a T_2_^*^ mapping sequence with an acceptable acquisition time and which is immune to system imperfections is challenging. T_2_^*^ mapping with radial sampling allows for better coverage of *k*‐space when imaging dynamic processes and is less sensitive to organ motion and blood flow artifact.^9^ In addition, undersampling of radial trajectories leads to incoherent aliasing, making them ideal for accelerated acquisitions with compressed sensing (CS)^10^ and parallel imaging (PI)^11^ reconstructions. However, radial trajectories are susceptible to errors arising from gradient delays and short‐term eddy currents,^12^ which could potentially be more pronounced on MR‐Linacs copared to conventional MRI scanners, because of the split‐gradient coil design and the large metal gantry that houses the linear accelerator. In particular, the split gradient coil of the high‐field MR‐Linac system used in this work has a large central gap (200 mm), which facilitates a less attenuated passage of the treatment beam, but could have implications on gradient performance and eddy current behaviours^2^ that impact *k*‐space trajectories.

Several approaches have been proposed to correct deviations of the gradient waveform. These include methods that require separate calibration scans^13^ or special hardware^14^, ^15^ to monitor the gradient field. Recently, accelerated acquisition of calibration data for fast R_2_^*^ and fat fraction quantification was proposed.^16^ In addition, calibration‐less methods were also developed, which include PI based techniques^17^, ^18^ and iterative approaches that jointly estimate images and gradient delays.^19^, ^20^ One such data‐driven method, called TRajectory Auto‐Corrected Image Reconstruction (TrACR), estimates trajectory errors from *k*‐space data by treating the gradient delay as an additional parameter in the objective function. TrACR can be applied to undersampled *k*‐space data through its seamless integration with PI techniques. Here, we extend this approach to include a T_2_^*^ relaxation term to enable gradient delay‐corrected T_2_^*^ mapping from fully and undersampled datasets acquired on an MR‐Linac. One key rationale behind using a data‐driven approach is that on MR‐Linac systems the position of the linac on the rotatable gantry can affect gradient delays, but information on the linac angle might not be available to the MR image reconstruction system, which renders prospective trajectory corrections necessarily incomplete. We evaluate the technique in a numerical phantom for different gradient delays and noise levels. Further, we assessed T_2_^*^ measurements in patients with prostate and head and neck cancer acquired during MR‐guided radiotherapy treatment workflows on an MR‐Linac.

## 2 MATERIALS AND METHODS

### 2.1 Theory

The proposed approach combines TrACR^19^ with model‐based reconstruction^21^ of the T_2_^*^ maps. The TrACR method is formulated as a joint estimation of images and *k*‐space trajectory errors, using an extension of the cost function for SENSE,^22^ where ∥.∥denotes the ℓ^2^ norm:

(1) $$\underset{\Delta k,x}{argmin}\sum_{n}^{N}\sum_{c}^{C}∥F_{n}\left(\Delta k_{n}\right)S_{c}x_{n}-y_{n,c}{∥}^{2}$$

Where $x_{n}\;$ is the series of images (*n* = 1, …, *N*, and *N* the number of echoes), $\left(\Delta k_{n}\right)$ is a vector of trajectory errors to be estimated, $F_{n}\left(\Delta k_{n}\right)\;$is the non‐uniform fast Fourier transform (NUFFT) operator for the *n* ^th^ echo parameterized by k‐space shifts caused by the delays ($\Delta k_{n}$) for every k‐space spoke, and $S_{c}\;$represents the coil sensitivity maps (with *c* = 1,…,C, and C the number of coils). The proposed approach extends the cost function in Equation (1) by including the physical model of *T*_2_^*^ relaxation $(M_{0}exp\left(-\frac{TE_{n}}{T_{2}^{*}}\right))\;$to regularize the cost function by enforcing model consistency.

(2) $$\begin{array}{ccc} & & \underset{\Delta k,T_{2}^{*},M_{0},x}{argmin}\sum_{n}^{N}\sum_{c}^{C}∥F_{n}\left(\Delta k_{n}\right)S_{c}x_{n}-y_{n,c}{∥}^{2}\\ & & \;+\;\lambda ∥x_{n}-{\hat{x}}_{n}\left(M_{0},\;T_{2}^{*}\right)\;{∥}^{2}\; \end{array}$$

Here $T_{2}^{*}\;$is the transverse relaxation time, **M**_0_ is the proton density map, $TE_{n}$ are the echo times, and λ is the regularization parameter. λ was chosen to be 1 to give equal weight to data and model consistency terms as mentioned in.^23^ The cost function in Equation (2) could be formulated such that the mono‐exponential decay model is introduced in the data‐consistency term and the **M**_0_, $T_{2}^{*}\;$parameters are individually regularized, however, minimizing such nonlinear equation can be numerically challenging and may lead to long reconstruction times. Therefore, in this work, the minimization problem was split into sub‐problems and was optimized in three alternating steps: trajectory correction, data consistency, and imposing model consistency. In the first step, $\Delta k_{n}$ is solved using the nonlinear conjugate gradient algorithm as described in.^19^ The second step involves enforcing the data consistency based on the updated $\Delta k_{n}$ in the NUFFT and obtaining the multi‐echo images $x_{n}$. As a third step, the magnitude of $x_{n}$ is updated by fitting a mono‐exponential decay $(|{\hat{x}}_{n}|=M_{0}exp\left(-\frac{TE_{n}}{T_{2}^{*}}\right))\;$on the magnitude images with a nonlinear least‐squares algorithm. The complex phase of ${\hat{x}}_{n}$ is preserved. Steps 1, 2, and 3 are iteratively repeated until the error between subsequent iterations is not decreasing significantly or the maximum number of iterations is reached. The initial guess for the image $x_{n}$ was obtained from the gridded reconstruction of the k‐space data $y_{n,c},\;$and $\Delta k_{n}$ was initialized with zeros. Coil sensitivities were calculated from the initial guess $x_{n}$ using the adaptive coil combination method.^24^

We compared three reconstruction methods to evaluate the performance of the proposed approach: (i) “uncorrected,” which corresponds to setting $\Delta k_{n}=\;0\;$and optimizing Equation (2) only with respect to $x_{n},\;M_{0,}\;\mathrm{and}\;T_{2}^{*},$ ^21^ (ii) “sequential,” corresponding to first solving for $\Delta k_{n}$ and $x_{n}$ using TrACR and then using nonlinear least squares fitting to estimate$\;M_{0}\;\mathrm{and}\;T_{2}^{*}\;,$and (iii) “joint” approach, where T_2_^*^ maps were jointly reconstructed with trajectory correction through alternating iterative reconstruction as described above.

The proposed approach was implemented in MATLAB 2019a (The Mathworks, Natick, Massachusetts, USA) on a desktop PC with an Intel Xeon E3‐1240 3.4 GHz CPU (Intel Corporation, Santa Clara, California, USA) and Nvidia RTX 6000 24 GB graphics card. All non‐uniform discrete Fourier transforms were computed using a GPU‐based non‐uniform fast Fourier transform (NUFFT) algorithm.^25^

### 2.2 Numerical simulations

Numerical simulations were performed on an anatomical brain phantom available from the Brain Web Simulated Brain Database.^26^ Simulations were based on a 256 × 256 brain image with T_2_^*^ relaxation and proton density maps. The ground truth image had different tissue types such as grey matter (GM), white matter (WM), cerebrospinal fluid (CSF), and skull. For the three main tissues, the following T_2_^*^ values were used: 84 ms for GM, 66 ms for WM, and 2000 ms for cerebrospinal fluid.^27^ Same T_2_^*^ values were used for all the pixels within each tissue types. Multi‐echo T_2_^*^‐weighted images were calculated using simulated coil sensitivity profiles for eight channels.^28^ The simulation was performed using a golden angle radial k‐space trajectory with 512 readout points and 402 radial spokes. Complex Gaussian noise with varying standard deviation was added to generate raw k‐space data of different noise levels (0, 0.1, 0.5, and 1) with the value of proton density fixed at 100. To undersample the dataset, 50% of the spokes were removed to achieve two times acceleration. In addition, two different undersampling schemes were tested. For the first undersampling scheme which will be referred to as “uniform,” spokes were removed from the same location for all echoes. Spokes were removed from the beginning of the acquisition to simulate a shortened acquisition while minimizing effects related to the system reaching steady‐state. For the second undersampling scheme called “alternate,” spokes were removed from a different location in the even and odd echoes. This is equivalent to using the first acquired spokes for all odd echoes and the last acquired spokes for all even echoes. For the used sampling protocol, this corresponded effectively to a rotation by ±33.02° between successive echoes. Gradient delays were simulated for x and y gradient coils separately ([1,−1] and [1, 2] in units of the sampling time), to generate corrupted k‐space data as described in.^20^ T_2_^*^ maps were reconstructed using the sequential and joint approach for fully sampled, uniform, and alternate undersampled data. Root mean square errors (RMSEs) with respect to the ground truth T_2_^*^ values were calculated for the reconstructed T_2_^*^ maps for sequential and joint approaches, for fully sampled and undersampled datasets and for different gradient delays. To calculate RMSEs, the skull ROI was excluded. In addition, relative error for each tissue type was calculated as: (abs [True T_2_^*^‐ calculated T_2_^*^]) / True T_2_^*^. The MATLAB code used for this can be found here (https://github.com/wbano1/Joint‐Gradient‐Delay‐T2‐Mapping).

### 2.3 Patient studies

In vivo data were collected in one prostate and one head and neck cancer patient undergoing radiotherapy on a 1.5 T MR‐Linac (Elekta AB, Stockholm, Sweden) within a study approved by the local research and ethics committee (PERMIT trial: NCT03727698). Written informed consent was obtained from all participants. The prostate cancer patient (male, 76 years) received radiotherapy after androgen deprivation therapy with a dose of 60 Gy/48.6 Gy to prostate and seminal vesicles in 20 fractions. The second patient (male, 66 years) had a base‐of‐tongue squamous cell carcinoma and received concurrent chemoradiotherapy with 65 Gy in 30 fractions. The T_2_^*^ mapping sequence was acquired twice per week during the “opportunity time,” which refers to the time during which daily adaptation, that is, contouring and treatment plan adaptation using an adapt‐to‐shape workflow^29^ is performed. The dataset was acquired using a radial stack‐of‐stars spoiled multi‐gradient echo sequence^30^ with the following parameters (8 echoes, 269 spokes, TR = 48 ms, ΔTE = 5 ms, FOV = 400 × 400 × 90 mm^3^, and 1.5 × 1.5 × 4 mm^3^ acquisition voxel size). Radial stack‐of‐stars sequence acquires radial spokes in the imaging plane whereas standard Cartesian phase encoding is performed in the slice direction. The radial spokes are rotated around the center by the golden angle, which results in cylindrical k‐space coverage. Considering the number of spokes, the data acquisition does not strictly fulfil the Nyquist criterion for reconstruction with the acquisition voxel size. The acquisition time for the fully sampled scan was 7:56 min. Raw data were exported from the scanner and T_2_^*^ maps were reconstructed off‐line. Trajectory correction was applied separately on even and odd echoes to consider the opposite readout polarity. The data was reconstructed and corrected for the gradient delays using the joint and sequential approaches for both fully sampled and undersampled datasets as described above for the simulated phantom and compared with uncorrected T_2_^*^ maps. An experienced radiologist delineated the tumor (T1), peripheral zone (PZ), and transition zone (TZ) based on the pre‐treatment images along with the planning target volume (PTV) and obturator internus muscle. In addition, a head and neck oncologist delineated PTV and parotid gland on the head and neck data. T_2_^*^ values from these delineations were compared for fully sampled and undersampled data sets.

### 2.4 Phantom experiment

To validate the proposed approach, a phantom experiment was performed where the ISMRM/NIST system phantom (CaliberMRI, Boulder, Colorado, USA) was scanned with both a radial and a Cartesian multi‐echo GRE sequence. In addition, a comparison to an established phase correction technique for radial MRI^12^ was performed. Details of the experiment and results are described in the supplemental material (Figure S1).

## 3 RESULTS

### 3.1 Simulation validation

**FIGURE 1:**
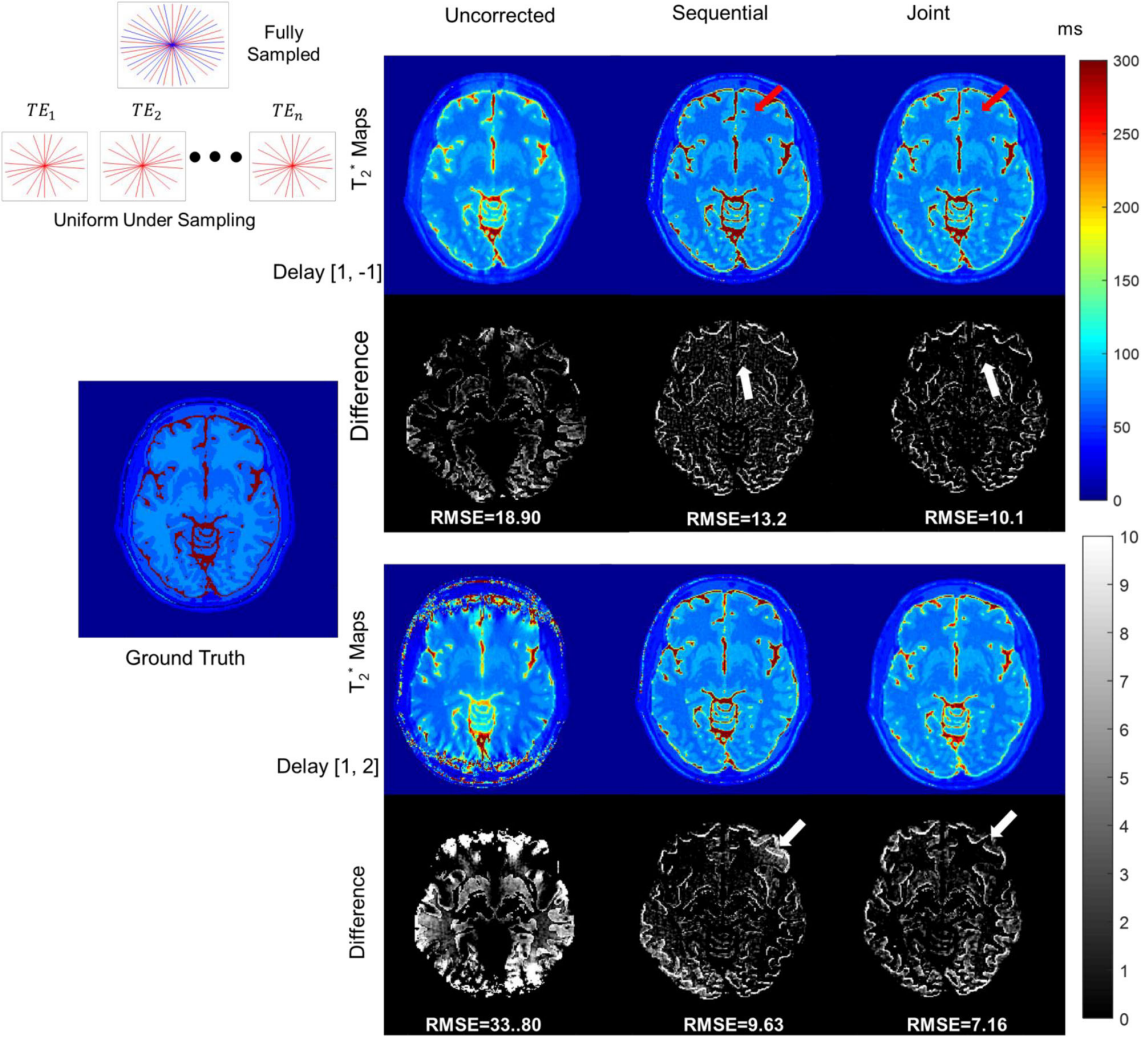
T_2_^*^ maps reconstructed from uniform undersampled (top left corner, identical radial angles for all echoes) numerical phantom data (noise level 0.1) for different gradient delays. Without any gradient delay correction, T_2_^*^ values in cerebrospinal fluid (CSF) are underestimated. The joint reconstruction outperforms the sequential and uncorrected approach in terms of RMSEs. For both gradient delays, T_2_^*^ maps reconstructed with the joint approach show less error (red arrows) that is more evident in absolute difference maps (white arrows) compared to the sequential approach. Color bars represent T_2_^*^ values in ms.

Figure 1 shows the results from numerical simulations for uniform undersampling with a gradient delay of [1, −1] and [1, 2] and the reconstructed T_2_^*^ maps without trajectory correction, and with trajectory correction using the sequential and joint approaches. For each gradient delay, the top row shows the reconstructed T_2_^*^ maps and the bottom row shows the absolute difference between ground truth and reconstructed T_2_^*^ maps. RMSEs are listed below each difference map. Uncorrected RMSEs for gradient delays [1, 2] are large when compared to [1, −1]. Difference maps show that joint estimation results in less error in the frontal lobe (white arrows) as compared to the sequential approach. T_2_^*^ maps reconstructed with the joint approach had less noise and lower RMSEs compared to the sequential approach.

**FIGURE 2:**
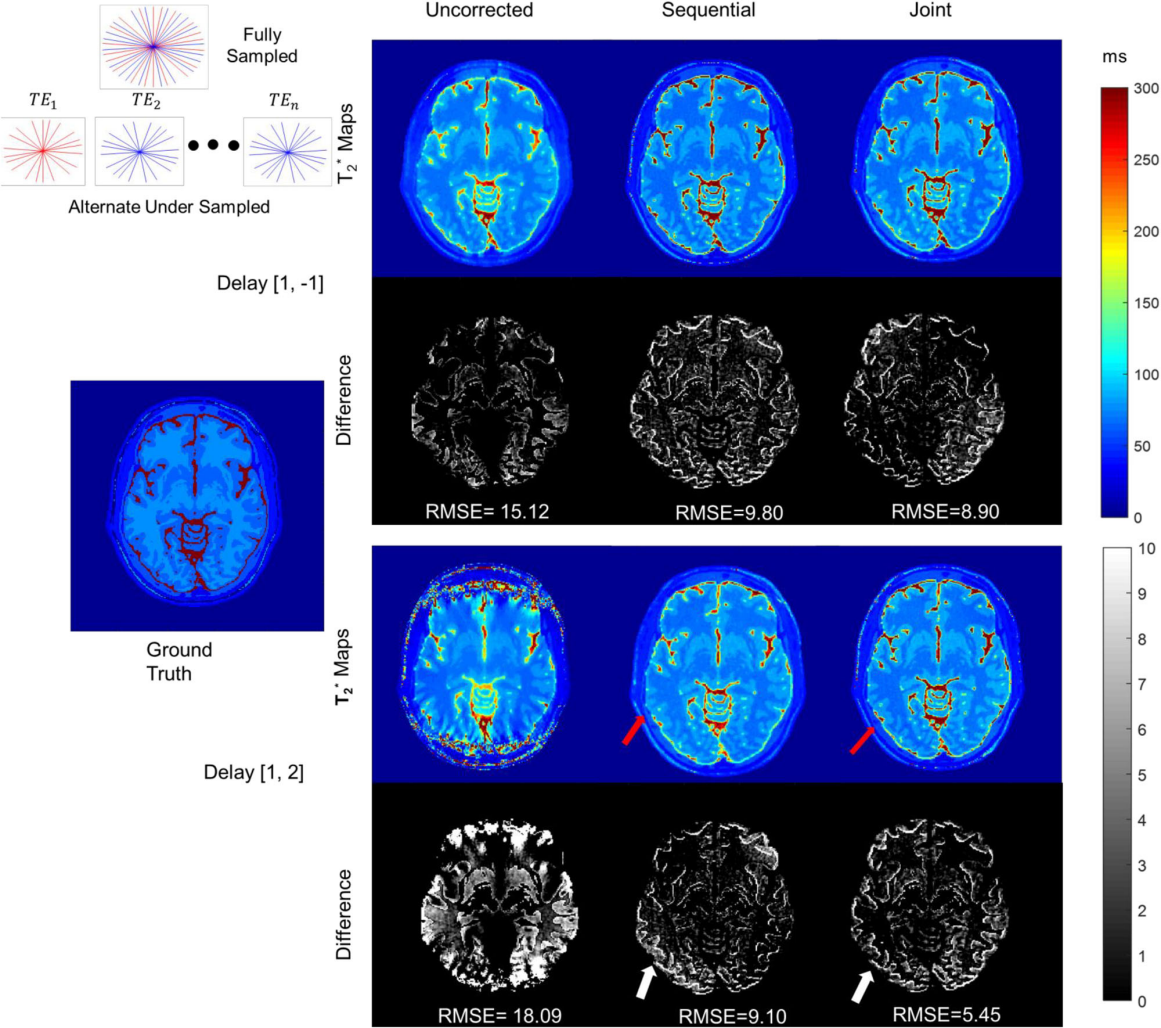
T_2_^*^ maps obtained from alternate undersampled numerical phantom data with a noise level of 0.1 for gradient delays [1, −1] (top) and [1, 2] (bottom). Fully sampled and alternate undersampling are shown in the top left corner, where even (red spokes) and odd echoes (blue spokes) have different spoke locations. Absolute difference maps show higher errors for T_2_^*^ maps reconstructed with the sequential approach (white arrows). RMSEs also show that T_2_^*^ maps reconstructed with the joint approach had less error than sequential or uncorrected maps. Colors represent T_2_^*^ values in ms.

Results from the alternate undersampling are shown in Figure 2. Overall, errors resulting from alternate undersampling were less as compared to uniform undersampling. For alternate undersampling and gradient delays [1, 2], T_2_^*^ maps reconstructed with the joint approach had less noise (red arrows) as compared to sequential approach.

Table 1 summarises the RMSE results for the numerical phantom experiments. For different noise levels, similar results were seen where the RMSEs increased at higher noise levels. For all noise levels, the proposed joint approach resulted in less error and lower RMSEs for both undersampling patterns and gradient delays.

**TABLE 1:** RMSEs of the reconstructed T_2_* maps in ms for both sequential and joint reconstructions of the numerical phantom data with different noise levels and gradient delays for uniform and alternate undersampling patterns.

RMSE (ms)	Gradient delay	Reconstruction	Noise levels			
			0	0.1	0.5	1
Uniform	[1 −1]	Sequential	12.31	13.01	13.92	15.23
		Joint	9.33	9.32	9.88	12.45
	[1 2]	Sequential	10.76	10.92	11.30	12.10
		Joint	5.74	5.89	5.87	6.01
Alternate	[1 −1]	Sequential	9.51	9.80	10.48	12.68
		Joint	8.76	8.90	9.55	10.76
	[1 2]	Sequential	9.06	9.10	9.77	11.64
		Joint	5.32	5.45	5.81	5.95

In all cases, the proposed approach outperforms a sequential correction. In all cases, the alternate undersampling scheme performs better than the uniform undersampling.

Similar results were observed for each tissue type (GM, WM, and CSF) in the numerical phantom where joint correction showed less relative error as compared to the sequential approach. Alternate undersampling also showed fewer relative errors as compared to uniform undersampling (supplementary material Figure S2). Artefact appearances for different gradient delays with and without correction are shown for coil‐combined images in supplementary Figure S3. For gradient delay [1,−1], artefacts appear predominantly as streaking artefacts, whereas additional signal voids are observed for the larger [1, 2] gradient delays. All these artefacts and distortions were effectively eliminated, and contrast was restored by the proposed method.

### 3.2 In vivo experiments

**FIGURE 3:**
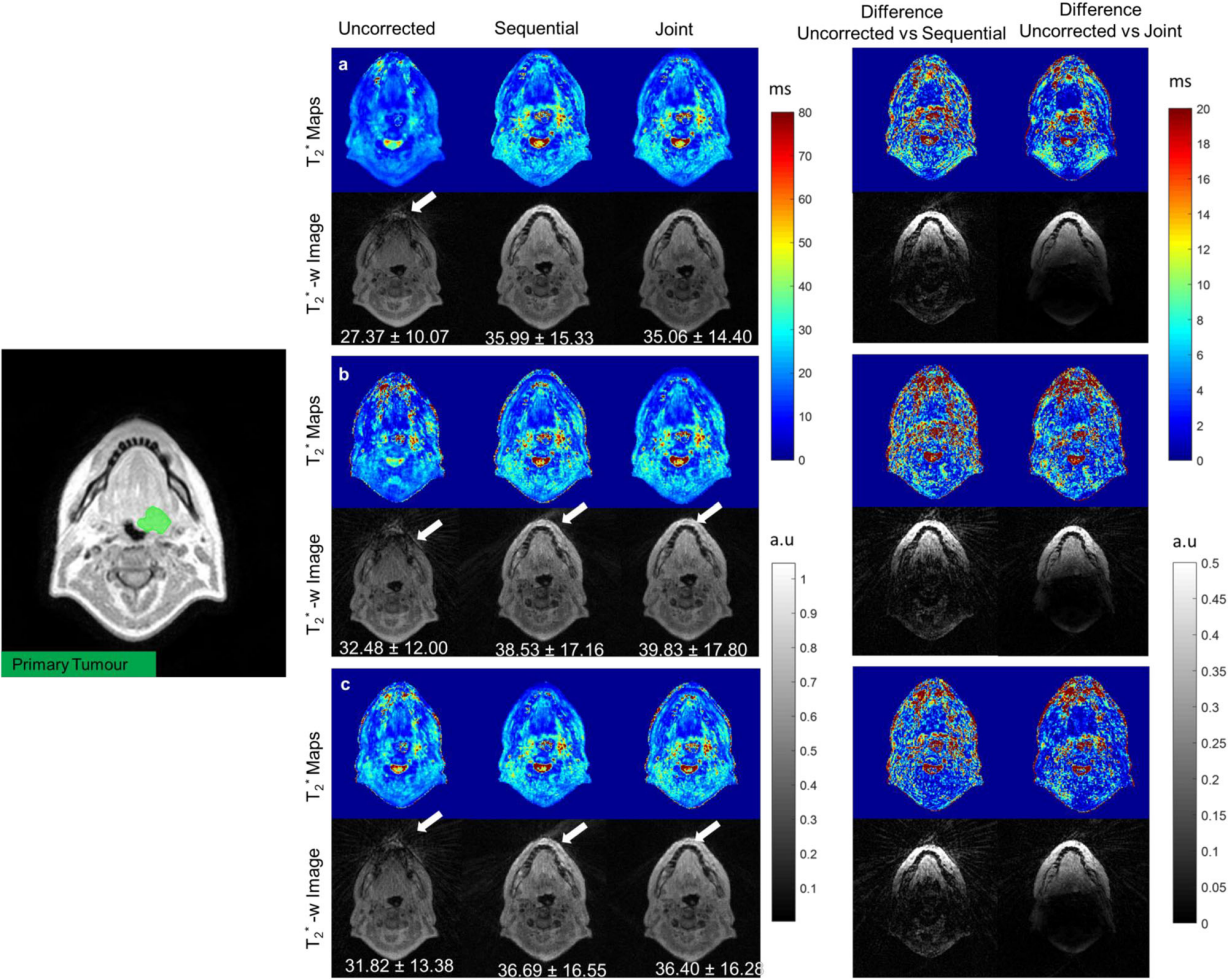
T_2_^*^ maps (Top) and T_2_^*^‐weighted images (TE = 5 ms) reconstructed from the head and neck cancer patient dataset: (a) fully sampled, (b) uniform undersampled, (c) alternate undersampled, without correction and with the sequential, respectively, joint approach. Mean T_2_^*^ values with standard deviations are mentioned for primary tumour. Absolute difference between T_2_^*^ maps and T_2_^*^‐weighted images reconstructed with and without trajectory correction are shown on the right. T_2_^*^‐weighted images reconstructed without trajectory correction show loss of signal and some streaking artefacts (white arrows). For the fully sampled dataset, both sequential and joint approaches removed these artefacts. Colors represent T_2_^*^ in ms and greyscale represents signal intensity in arbitary units.

**FIGURE 4:**
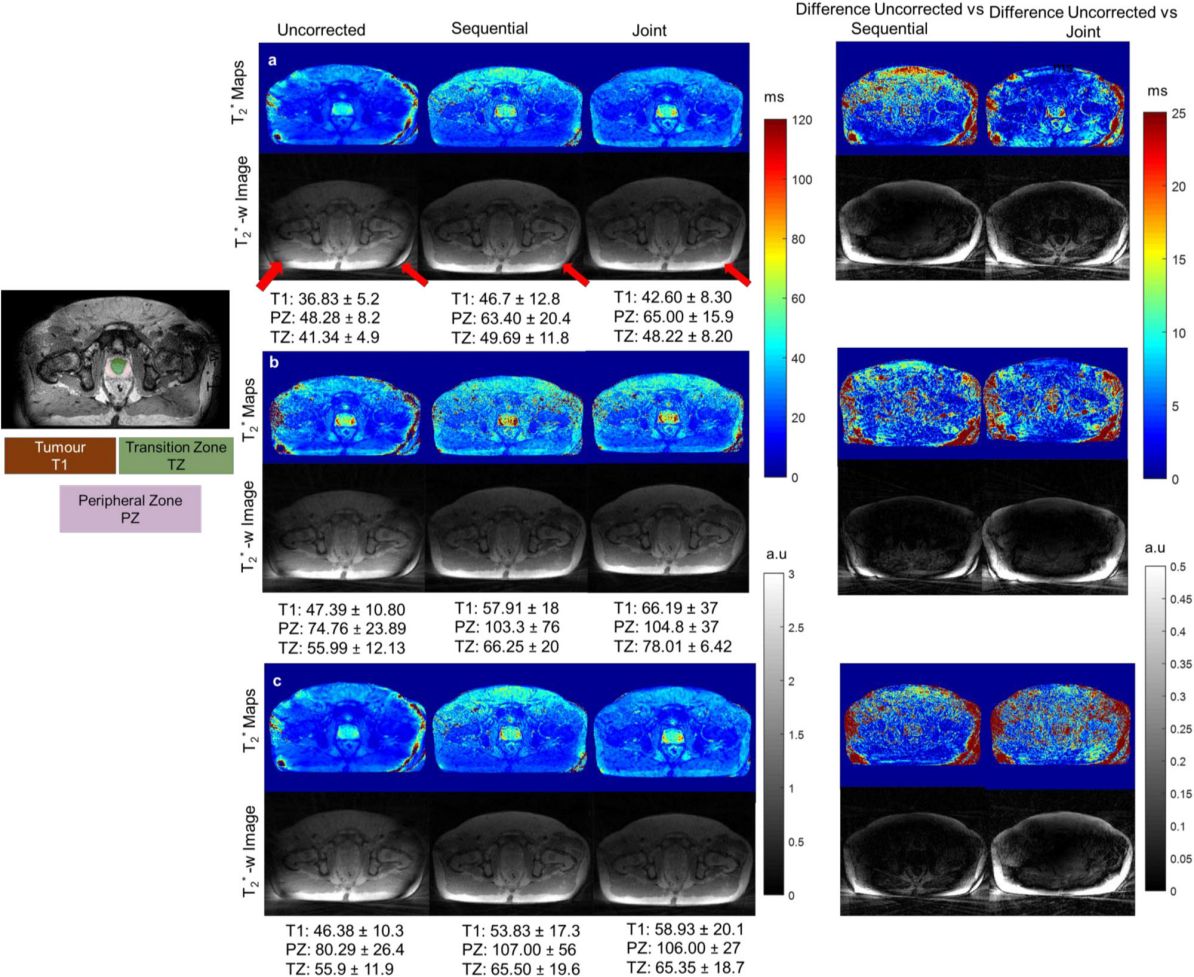
T_2_^*^ maps (Top) and T_2_^*^‐weighted images (TE = 5 ms) reconstructed from the prostate cancer patient dataset: (a) fully sampled, (b) uniform undersampled, (c) alternate undersampled without correction and with the sequential, respectively, joint approach. Mean T_2_^*^ values with standard deviations are mentioned for tumor (T1), peripheral zone (PZ), and transition zone (TZ). Absolute differences between T_2_^*^ maps and T_2_^*^‐weighted images reconstructed with and without trajectory correction are shown on the right. Arrows highlight a banding artifact, which disappeared on one side after trajectory correction, but partially remained on the other side. Colors represent T_2_^*^ in ms and greyscale represents signal intensity in arbitary units.

The proposed method was used to reconstruct T_2_^*^ maps from one prostate and one head and neck cancer patient. T_2_^*^ maps and reconstructed T_2_^*^‐weighted images (TE = 5 ms) from fully sampled and undersampled datasets and reconstructed with sequential and joint approaches are shown in Figures 3 and 4. Artefacts resulting from gradient delays can be seen in the head and neck and prostate (white arrows) T_2_^*^ maps and the T_2_^*^‐weighted images. Both sequential and joint reconstructions reduced these artefacts, and the image quality was improved visibly. Additionally, the difference images between the uncorrected and corrected T_2_^*^ are shown to demonstrate the performance of the proposed method. For the prostate patient, T_2_^*^ values within the prostate are higher compared to the uncorrected maps for both sequential and joint approaches.

**FIGURE 5:**
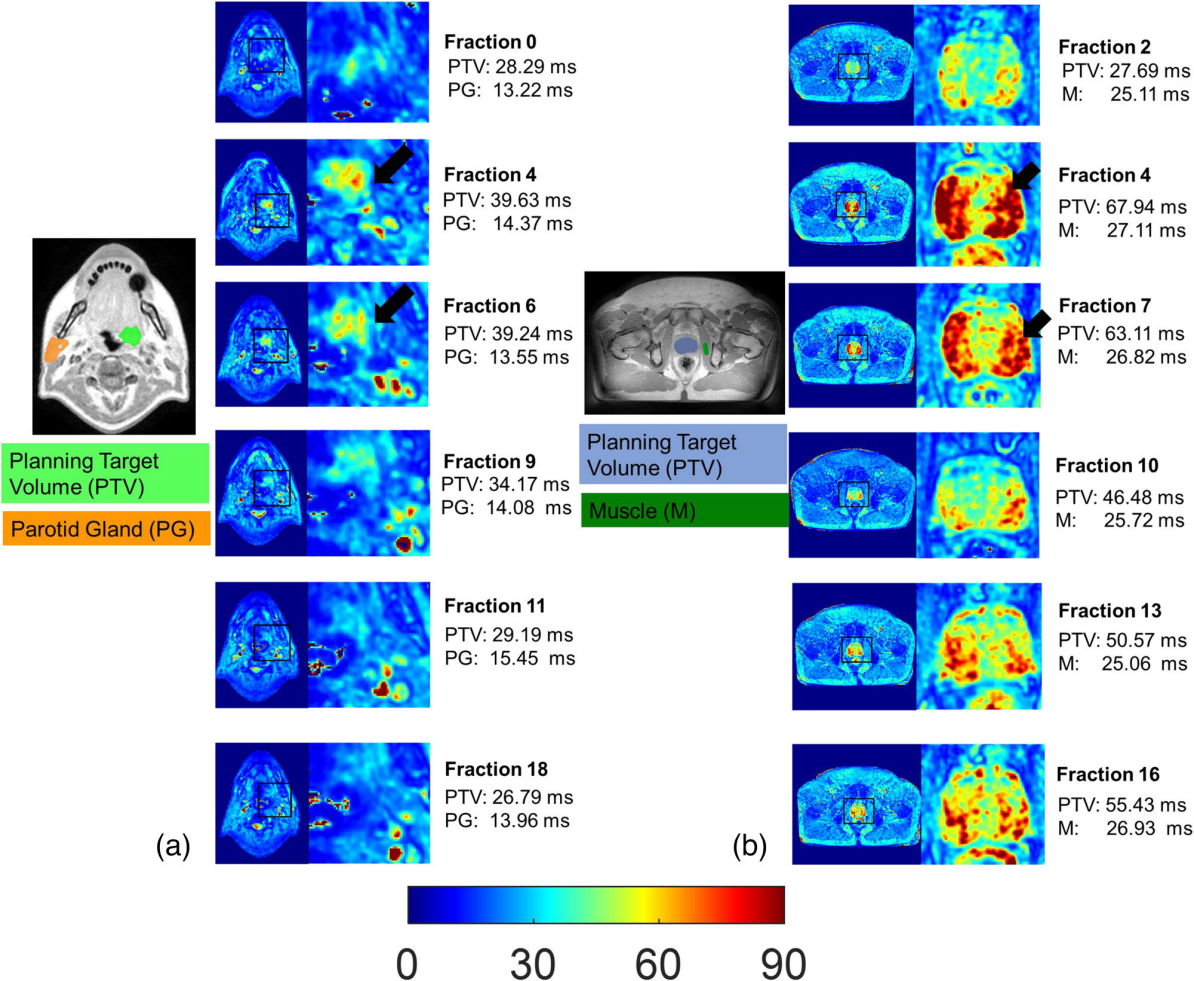
T_2_^*^ maps from different fractions over the course of radiotherapy treatment with corresponding planning tumor volume (PTV) shown overlaid with a T_2_^*^‐weighted image (TE = 5 ms) on the left. Fraction 0 is the first fraction when T_2_^*^ maps were acquired whereas subsequent fraction numbers are relative to the first fraction at which T_2_^*^ maps are acquired. (a) There is an increase in mean T_2_^*^ value within PTV in fraction 4 and 6 (arrows) that subsequently decreased until fraction 18. Tumor was completely resolved at the end of treatment. Mean T_2_^*^ values within the parotid gland were stable throughout treatment. (b) For the patient with prostate cancer, mean T_2_^*^ values showed a more complex behavior, increasing substantially until fraction 7 (arrows), then dropping around fraction 10 and increasing again until the end of treatment. Overall, values within muscle showed less variation. This increase in T_2_^*^ values could be indicative of fibrosis or apoptotic changes in the prostate.

Figure 5 shows the T_2_^*^ maps reconstructed with the proposed approach from the fully sampled undersampled dataset for head and neck and prostate cancer patients for different treatment fractions. The variability and the changes in the T_2_^*^ values within the PTV can be seen visually in the corrected T_2_^*^ maps across different fractions and is related to daily variations of the pelvic anatomy due to changes in the filling of hollow organs, such as the rectum.^31^

## 4 DISCUSSION

Imaging tumor hypoxia non‐invasively could be used to map the spatial distribution of potential radio‐resistant regions before and during the course of radiotherapy and adapt the treatment to overcome resistance.^32^ Hypoxia imaging with oxygen‐enhanced MRI and R_2_^*^ mapping was demonstrated previously and could be incorporated into routine MR‐based treatment planning.^33^ However, the potential to characterize hypoxia with T_2_^*^ mapping on a hybrid MR‐Linac has not been studied before. Bi‐parametric (T_2_ and diffusion‐weighted imaging) based tumor boosting has already been shown to improve biochemical relapse‐free survival at conventional fractionations for prostate.^34^, ^35^ Incorporating hypoxia imaging into the daily MR‐Linac treatment workflow could facilitate “dose painting” approaches,^36^ where dose prescriptions are adapted to the tumor's spatial micro‐environment.

In this proof‐of‐concept study, we demonstrated the feasibility of T_2_^*^ mapping for MR‐guided radiotherapy. We found that *k*‐space trajectory errors must be corrected since they affect the accuracy of the T_2_^*^ estimates on MR‐Linacs. Regarding accelerated MR image acquisitions, gradient delay estimation was previously investigated using iterative PI‐based methods that exploit correlations in the receive channels to correct for gradient delays. Deshmane et al. used GRAPPA operator gridding to obtain a trajectory estimate and a corrected radial k‐space signal.^17^ A low‐rank and PI based method was also proposed to simultaneously estimate gradient delays and coil sensitivities.^20^ In addition, a general framework for radial trajectory correction and image reconstruction (TrACR) was proposed.^19^ Alternatively, calibration scans could be utilized to fully characterize the gradient impulse response function (GIRF) of the scanner.^15^, ^37^, ^38^ However, GIRF‐based methods cannot capture short‐term gradient variations, for example, due to heating of the gradient coil.^39^ In comparison to these methods, our approach includes the physical model of T_2_^*^ relaxation to better regularize the cost function and thereby improve the image reconstruction and trajectory correction. A key advantage of our approach is that gradient delays can be corrected for without the need for specific calibration scans. It could be used in multicenter studies, where gradient delays differ between individual installations, for example due to differences in hardware or software versions. In the particular context of MR‐Linac systems, our approach could be applied independently of the position of the linac on the rotating gantry, which could affect the eddy current behavior of the imaging gradient system^40^ and has an influence on field homogeneity.^41^

With the help of numerical simulations and in vivo experiments, we demonstrated that our proposed approach can correct *k*‐space trajectory errors in radial acquisitions to result in more accurate T_2_^*^ estimates. Our simulations demonstrated that for different noise levels and undersampling patterns, the proposed approach resulted in fewer errors compared to a sequential correction technique. Incorporating TrACR corrections led to significant visible improvements (reduced streaking and banding artefacts) over uncorrected images in the in vivo experiments.

One potential advantage of radial trajectories is that undersampling leads to incoherent aliasing, which can be exploited by PI or CS reconstructions. In addition, radial sampling can be used to capture dynamic processes and has been used specifically for free‐breathing motion‐resolved R_2_^*^ mapping.^42^, ^43^ Spatial regularization in the form of a sparsifying transform could be used to improve the reconstruction of the undersampled data.^44^ Such CS type reconstructions^45^, ^46^ rely on sampling patterns that result in incoherent aliasing. In the current work, we explored two different sampling schemes to identify whether varying the angular sampling pattern for different echo times, as suggested in^47^ leads to further improvements of the image reconstruction. The alternate undersampling scheme used in this work promotes incoherent aliasing and has the additional advantage of maintaining the golden angle ratio individually for the sampling of each echo, thereby inheriting its near‐optimal properties.^48^ Future work will explore prospective undersampling of in vivo data with alternating or rotating sampling patterns. This could allow further acceleration of data acquisition or lead to improved quality of the reconstructed T_2_^*^ maps.

The role of T_2_^*^ in measuring hypoxia is disputed as the absolute value is dependent on many factors such as vascular oxygenation level, T_2_ relaxation,^7^, ^49^ blood volume fraction,^50^ and macroscopic magnetic field inhomogeneities.^51^ Hence, decoupling of blood flow volume and deoxyhemoglobin is needed to indicate the actual oxygenation. Previous studies have addressed this challenge by modelling these factors with T_2_^*^ to obtain estimates of oxygenation concentration.^52^, ^53^, ^54^ In this context, correct estimates of T_2_^*^ with gradient delay correction could be combined with these methods for better characterization of hypoxia. In the context of hypoxia, functional imaging (including T_2_^*^ mapping, diffusion‐weighted imaging (DWI), oxygen‐enhanced imaging) can be used to detect cyclic hypoxia that can last few minutes^3^ to chronic hypoxia lasting weeks.^55^ In addition, tumor R_2_^*^ has shown to be a prognostic indicator of acute radiotherapeutic response in tumors.^56^ In addition, first in vivo efforts have been made to implement hypoxia imaging on MR‐Linacs for head and neck cancer.^57^ Implementing these methods on MR‐Linacs is challenging, so initial feasibility studies such as the work presented herein, are of great value. We showed that T_2_^*^ mapping can be integrated into clinical treatment workflows on an MR‐Linac. This could aid the design of future clinical trials that would base biologically adaptive radiotherapy on T_2_^*^ mapping to overcome resistance.

One current limitation of our un‐optimized prototype reconstruction is the long computation time (22 min). This could hinder the use of T_2_* maps reconstructed with the proposed approach for online adaptive treatment adaptation on MR‐Linacs, where immediate reconstruction of the T_2_^*^ maps would be required. Future work will explore acceleration of the reconstruction, for example, by employing machine learning‐based methods. Another limitation of our work is that the radial acquisition used in the in vivo experiments did not fulfil the Nyquist criterion in the k‐space periphery, and due to acquisition time constraints, we were not able to additionally include a Cartesian T_2_* mapping sequence for comparison. T_2_^*^ values in the peripheral zone of prostate at 1.5 T as reported by Alonzi et al.^58^ are 67 ms as compared to 65 ms (for fully sampled radial with gradient delay correction) in the present work. In addition, field inhomogeneity and motion correction were not considered in this work. Future work will explore whether these will improve the robustness of the technique and, potentially, extend the technique to treatment sites impacted by physiological motion.

## 5 CONCLUSION

In this proof‐of‐concept study, we explored the feasibility of fast T_2_^*^ mapping and its incorporation into the clinical treatment workflow. We found that joint trajectory correction with model‐based reconstruction improved the quality of T_2_^*^ maps for fully and undersampled data sets. Our proposed technique decreased scan time by 50% (to 3.5 min), which minimizes the burden for patients and facilitates incorporation of additional qMRI techniques into MR‐guided radiotherapy workflows on MR‐Linac systems.

This project represents independent research supported by the National Institute for Health research (NIHR) Biomedical Research Centre at The Royal Marsden NHS Foundation Trust and the Institute of Cancer Research, London. The views expressed are those of the authors and not necessarily those of the NIHR or the Department of Health and Social Care.

## CONFLICT OF INTEREST STATEMENT

The Institute of Cancer Research and the Royal Marsden NHS Foundation Trust are members of the Elekta MR‐Linac research consortium. Uwe Oelfke and Alison Tree receive research funding from Elekta.

## Supporting information

Supplementary information
