# Supplementary material for: Joint radial trajectory correction for accelerated T2 * mapping on an MR‐Linac
Source: Med Phys. 2023 May 27;50(11):7027–38. doi: 10.1002/mp.16479 (PMC10946747; doi:10.1002/mp.16479)
Supplement: Supplementary file 1 — Supplementary information [file MP-50-7027-s001.pdf]

Supplementary Material S1:

Phantom Experiment:

An ISMRM/NIST phantom (Calibre MRI, Boulder, CO, USA) was scanned using a radial stack-of-stars spoiled multi-gradient echo sequence with the following parameters (8 echoes, 269 spokes, TR = 48 ms, ΔTE = 5 ms, FOV = 400x400x90 mm^3 and 1.5x1.5x4 mm^3 acquisition voxel size). The acquisition time for the fully sampled scan was 7:56 minutes. For comparison, a fully sampled 3D Cartesian GRE sequence was acquired with the same parameters and acquisition time of 7:46 minutes. For Cartesian acquisitions, DICOMs were exported and T_2^* maps were calculated from the magnitude images using a non-linear least squares algorithm. For the radial dataset, raw data were exported from the scanner and T_2^* maps were reconstructed offline using the joint and sequential approaches to gradient delay correction as described in the main manuscript. For additional comparison, T_2^* maps were reconstructed using a phase correction method for radial MRI[1]. T_2^* maps were reconstructed for both fully sampled and undersampled datasets as described for the numerical simulations and compared with Cartesian T_2^* maps. Region of interests (ROIs) were drawn within all compartments and mean T_2^* values for Cartesian, fully sampled and undersampled data were compared for uncorrected, sequential and joint approaches.

Results:

Figure S1 shows a comparison of the Cartesian and radial T_2^* maps obtained from the ISMRM/NIST phantom experiment. For radial datasets, T_2^* values for all compartments were lower when compared to Cartesian data in both fully sampled and undersampled cases. Relative difference was calculated between Cartesian T_2^* maps, and reconstructed T_2^* maps with four approaches (uncorrected, sequential, phase correction and joint). Relative differences for sequential, joint and phase-corrected fully sampled dataset showed that joint reconstruction resulted in less error as compared to sequential and phase correction reconstruction (Figure S1, panel f).

It should be noted that Cartesian and fully sampled radial with the same acquisition time are not equivalent. To achieve equivalent k-space coverage, radial sampling requires roughly π/2 times the number of Cartesian lines and even then, the point-spread functions are still different. Additionally, B_0 inhomogeneity affects radial and Cartesian trajectories differently, leading to blurring for the radial and image shifts for the Cartesian trajectories, which could explain some of the observed differences. This is relevant for the used MR-Linac system because only first-order shimming was available. In particular, we noted that the signal intensity for the Cartesian T_2* mapping sequence fluctuated between odd and even echoes. For vial 12 the goodness of fit for the Cartesian sequence was only R^2=0.824 vs R^2=0.994 for the radial sequence evaluated on the images reconstructed on the MR console.

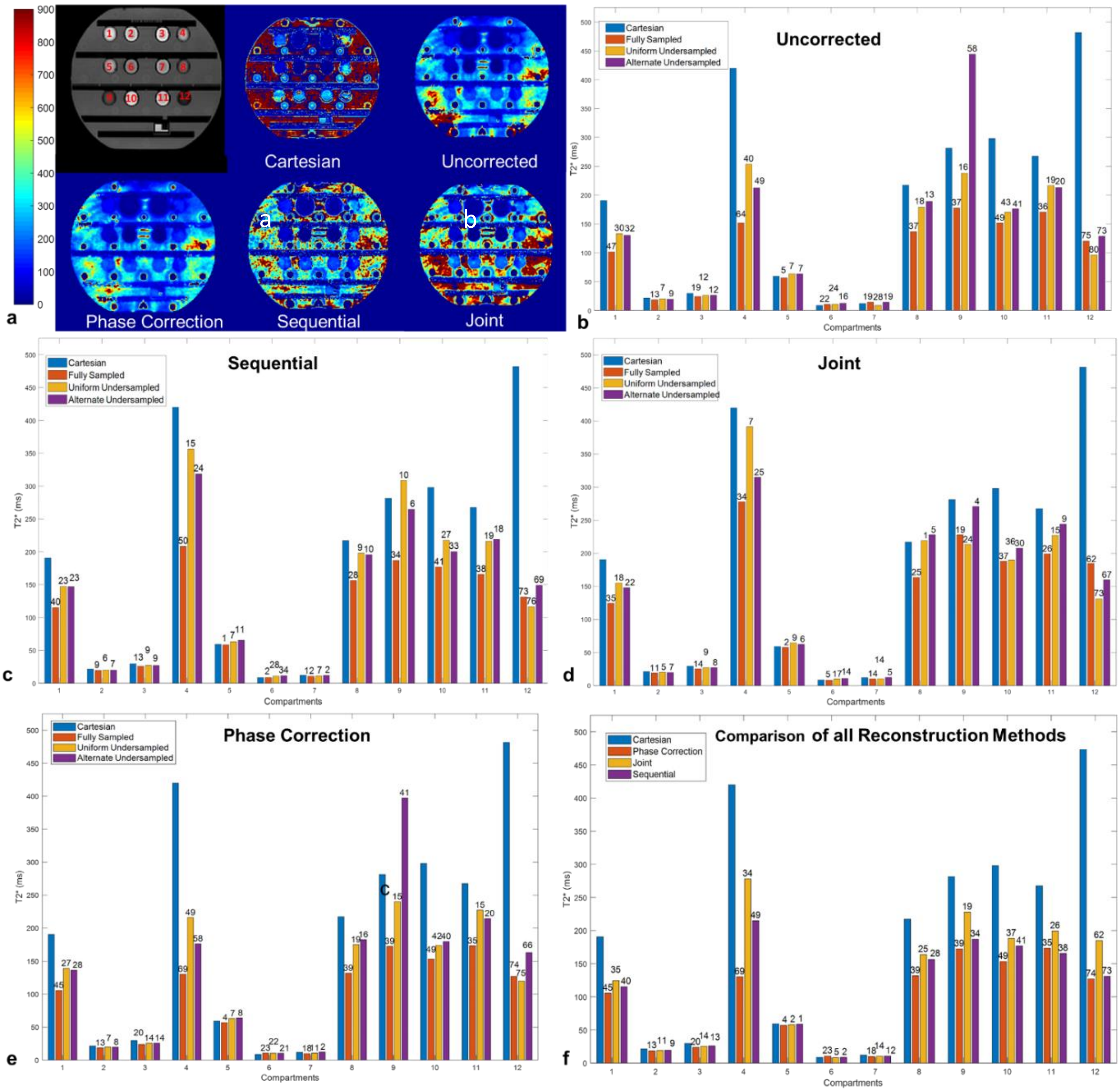

Figure S1: (a) T_2* maps from NIST/ISMRM phantom for Cartesian data, fully sampled without gradient delay correction, sequential and joint approaches. ROI analysis comparing T_2^* values for (b) uncorrected, (c) sequential (d) joint approaches (e) phase correction and (f) comparison of all reconstruction methods for fully sample dataset. Relative difference in % with respect to the Cartesian value is mentioned above each bar. T_2^* values calculated from radial data were in general lower than those obtained with Cartesian sampling. T_2^* values calculated using both sequential and joint approaches had lower relative differences compared to the uncorrected and phase corrected T_2^* values. Large differences were found for vial 12, which could be due to the relatively low signal in that vial.

1. Moussavi A, Untenberger M, Uecker M, Frahm J. Correction of gradient-induced phase errors in radial MRI. *Magnetic resonance in medicine.* 2014;71(1):308-312.

Supplementary Material S2:

Relative absolute errors for grey matter (GM), white matter (WM) and cerebrospinal fluid (CSF) regions in the numerical phantom for the noise level 0.1 calculated as (abs (True T_2^*- calculated T_2^*)) / True T_2^* below in Table 1a and 1b. Results showed that the relative error of the joint reconstruction with alternate undersampling was less than for the sequential approach for all three tissue types. Relative errors in CSF are very high, because the probed echo times (up to 40 ms) are very short compared to the assumed ground truth value of 2000 ms.

Results:

Tissue Type	Undersampling	Absolute relative error (%)		
		Uncorrected	Sequential	Joint
GM	Uniform	31.20	12.76	11.77
	Alternate	12.87	10.86	9.43
WM	Uniform	3.10	2.40	1.99
	Alternate	3.00	1.36	1.32
CSF	Uniform	74.37	60.19	57.05
	Alternate	74.16	57.75	53.68

Table S2a: Absolute relative error (in %) for grey matter (GM), white matter (WM) and cerebrospinal fluid (CSF) for gradient delay [1, -1] and noise level 0.1.

Tissue Type	Undersampling	Absolute relative error (%)		
		Uncorrected	Sequential	Joint
GM	Uniform	12.68	10.77	8.44
	Alternate	11.70	4.98	2.30
WM	Uniform	13.63	4.93	1.66
	Alternate	12.69	2.11	1.51
CSF	Uniform	80.79	58.20	73.36
	Alternate	80.61	48.91	72.36

Table S2b: Absolute relative error (in %) for grey matter (GM), white matter (WM), and cerebrospinal fluid (CSF) for gradient delay [1, 2] and noise level 0.1.

Supplementary Material S3:

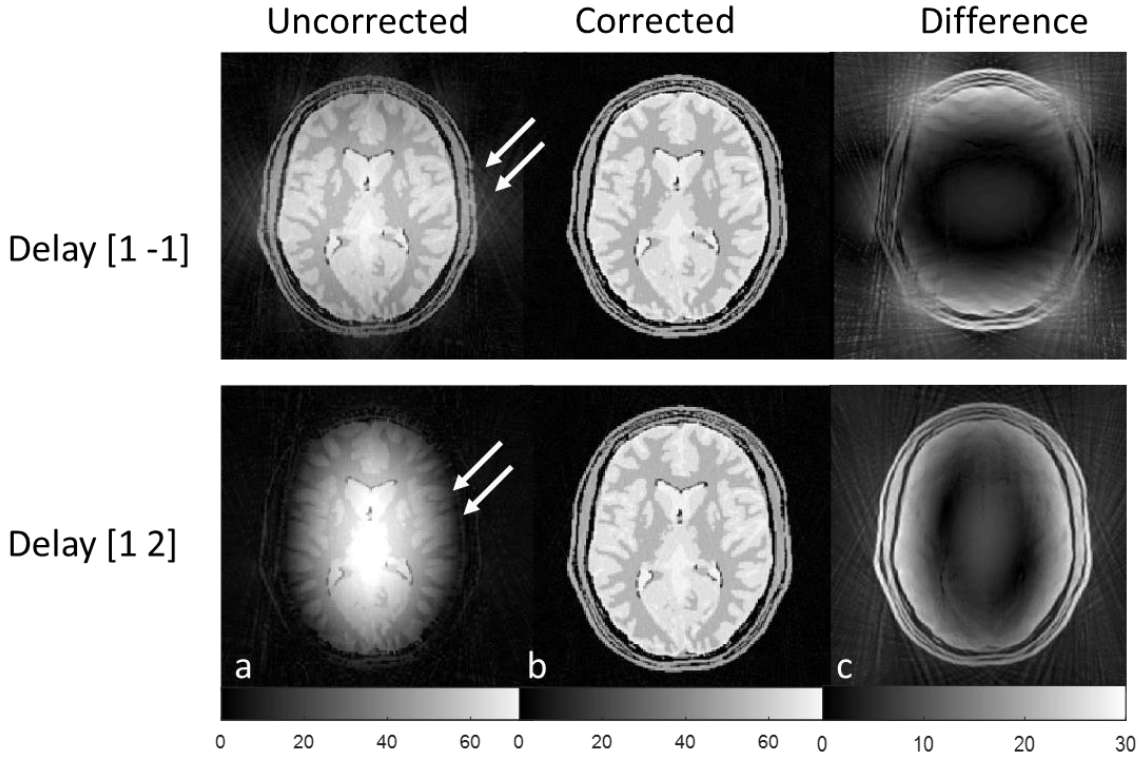

Figure S3: T_2^* weighted image from numerical phantom (TE = 40 ms) with different gradient delays (a), corrected with joint trajectory correction (b) and difference image (c). Arrows indicate the artefacts appearing in the form of streaking (top) or contrast variation(bottom). All artefacts indicated by arrows were effectively eliminated with the proposed approach considerably improving the image quality.
